# Synergistic Effect of BDNF and FGF2 in Efficient Generation of Functional Dopaminergic Neurons from human Mesenchymal Stem Cells

**DOI:** 10.1038/s41598-017-11028-z

**Published:** 2017-09-04

**Authors:** Manisha Singh, Anupama Kakkar, Rinkey Sharma, O. P. Kharbanda, Nitika Monga, Manish Kumar, Shantanu Chowdhary, Balram Airan, Sujata Mohanty

**Affiliations:** 1 Stem Cell Facility (DBT- Centre of Excellence for Stem Cell Research), All India Institute of Medical Sciences, New Delhi, India; 2 Department of Orthodontics and Dentofacial Deformities, Centre for Dental Education and Research (CDER), All India Institute of Medical Sciences, New Delhi, India; 3 Institute of Genomics and Integrative Biology, New Delhi, India; 4 Department of Cardiothoracic and Vascular Surgery, All India Institute of Medical Sciences, New Delhi, India

## Abstract

To understand the process of neurogenesis, generation of functional dopaminergic (DAergic) neurons from human mesenchymal stem cells (hMSCs) is important. BDNF has been reported to be responsible for inducing neuronal maturation and functionality. Previously, we have reported the efficient generation of neurons from human bone marrow derived MSCs using FGF2 alone. We hypothesize that hMSCs from various tissues [(bone marrow (BM), adipose tissue (AD) and dental pulp (DP)], if treated with BDNF on 9^th^ day of induction, alongwith FGF2 will generate functional DAergic neurons. Hence, cells were characterized at morphometric, transcription and translational levels for various markers like MAP2, TH, NGN2, PITX3, DAT, synaptophysin, Kv4.2 and SCN5A. Functionality of *in vitro* generated neurons was studied by calcium ion imaging. Result analysis depicted that BDNF has effect on expression of dopaminergic neuronal markers at gene and protein levels and functionality of neurons. Among these hMSCs, DP-MSC showed significantly better neuronal characteristics in terms of morphology, expression of neuronal markers and foremost, functionality of neurons. From the present study, therefore, we concluded that i) BDNF has additive effect on neuronal characteristics and functionality ii) DP-MSC are better MSC candidate to study DAergic neurogenesis and perform future studies.

## Introduction

Neurogenesis is defined as the process of formation of nerve cells or neurons from their progenitor cells. The ability of adult vertebrate brain to form neurons is restricted to specific regions only, like subgranular zone of the hippocampal dentate gyrus and rostral parts of the lateral ventricles of the subventricular zone^1^. Neurogenesis is also believed to occur in cerebral neocortex region^2^. It is important to study the process of neurogenesis to get the actual sequence of events occurring *in vivo*, which will further help in developing strategies for drug delivery system or cell based therapy. Apart from being more suitable candidate for clinical trials than embryonic stem cells (ESC), adult stem cells (ASC) obtained from tissues like bone marrow (BM), adipose tissue (AD), dental pulp (DP) and Wharton’s jelly (WJ) are also being used as cell models for various kinds of *in vitro* studies^3–7^.

Over a period of time, several research groups have reported the *in vitro* differentiation of human Mesenchymal Stem Cells (hMSCs) into neuronal cells, using various strategies like chemicals, growth factors, conditioned media, co- culture, direct genetic programming, differentiation from induced pluripotent stem cells and by using scaffolds to mimic the matrix^8–17^. However, there are very few reports targetting *in vitro* generation of dopaminergic neurons^18–21^^.^

Dopaminergic (DAergic) neurons are the sub- specification of neurons which are capable of secreting dopamine and help in neuro- muscular coordination. Degeneration of DAergic neurons is associated with the onset of Parkinson’s disease. Hence, understanding the genesis of DAergic neurons will help in devising drug testing cell models or stem cell based treatment regimes in future. Various growth factors such as FGF2, FGF8, SHH, BDNF and all- trans retinoic acid (ATRA) have been used widely to generate DAergic neurons fron MSC. In our recently published study^20^, we have reported the induction of dopaminergic (DAergic) phenotype in BM-MSC using only FGF2. The DAergic neurons derived from BM-MSC showed expression of DA- specific marker, tyrosine hydroxylase (TH) with all the induction cocktails. However, the electrical functionality of the neurons was not well studied. Detailed literature search was conducted to find out the vital factors responsible for functional maturity of neurons. Addition of BDNF to the induction medium is reported to increase the number of functional neurons^18^ hence, we aimed to evaluate and compare the differential effect of BDNF in inducing the functionality in DAergic neurons generated from hMSCs. After induction with both the protocols, i.e., with and without BDNF, we have characterised the cells for their neuronal markers specific to DAergic neurons and studied their functionality. After induction with both the protocols, presence of cell types other than DAergic nenurons, like glial cells, serotonergic neurons, cholinergic neurons and schwann cells was also investigated. In this study, we have also compared the response of hMSCs obtained from different human tissue sources (BM, AD and DP) towards the two protocols of dopaminergic neuronal induction.

## Results

Cryopreserved BM-MSC, AD-MSC and DP-MSC were revived, expanded, characterized and evaluated for their proliferation. It was observed that all the hMSCs had comparable MSC characteristics, as depicted by flow cytometric profiling and trilineage differentiation (Supplementary Fig. 1). However, proliferation studies suggest that population doubling time of DP-MSC is lesser than that of BM-MSC and AD-MSC hence, seconding the observations of MTT assay that proliferation rate of DP-MSC is higher than that of other two hMSC counterparts (Supplementary Fig. 2).

### DP-MSC prove to be better cell model to study the *in vitro* generation of cells of neuronal lineage while, BM-MSC and AD-MSC fair equivalent

#### hMSCs acquire neuronal cells like morphology during the induction period

**Figure 1:**
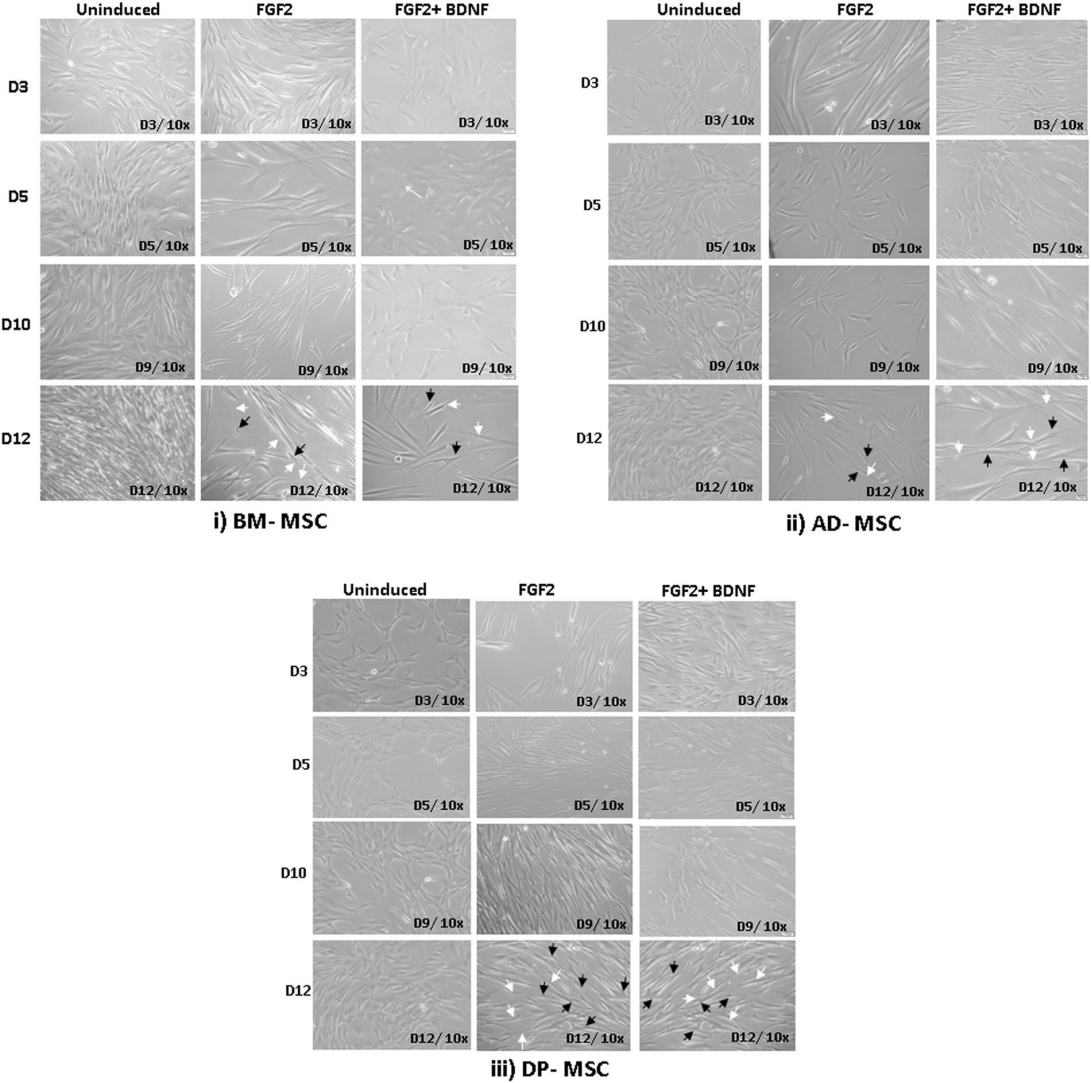
Morphological changes in hMSCs during various time points (Day 3, day 5, day 10 and day 12) of neuronal induction. (i) BM-MSC; (ii) AD-MSC; (iii) DP-MSC. The morphology of hMSCs have changed from spindle shaped to perikaryl. Black arrows show distinct cell bodies and white ones represent axon and dendritic structures emerging from the axon hillock part (V-shaped) of the cell body. Appearance of neuronal morphology start appearing from 6–7^th^ day of induction. The dimensions of the neuronal cells are similar to those observed in native neuronal cells, as discussed later. Single cells are shown to elaborate the morphological features of the differentiated hMSCs.

During the induction period, the cytoplasm in the flat spindle shaped cells initially retracted towards the nucleus and formed a contracted multipolar cell body, which left membranous processes much like peripheral extensions over the subsequent days of induction period. The cell bodies (CB) became increasingly spherical, retractile, and exhibited a typical neuronal perikaryal appearance. Also, axon hillock- like structures were observed around the cell body from where the neurites-like structures were found emerging. Some of the cells had multiple neurites (2–6), while others had tridentate structure. In most of the other cells, they had neucleus shifted towards one end, but having bipolar morphology only (Fig. 1). These morphological features started appearing on 6–7^th^ day of inducton. After observing these morphological features during the induction period, we performed detailed analysis, as reported in next section of results.

### Neuritogenesis and axonogenesis occurred better in DP-MSC alongwith morphological changes than in BM-MSC and AD-MSC

**Figure 2:**
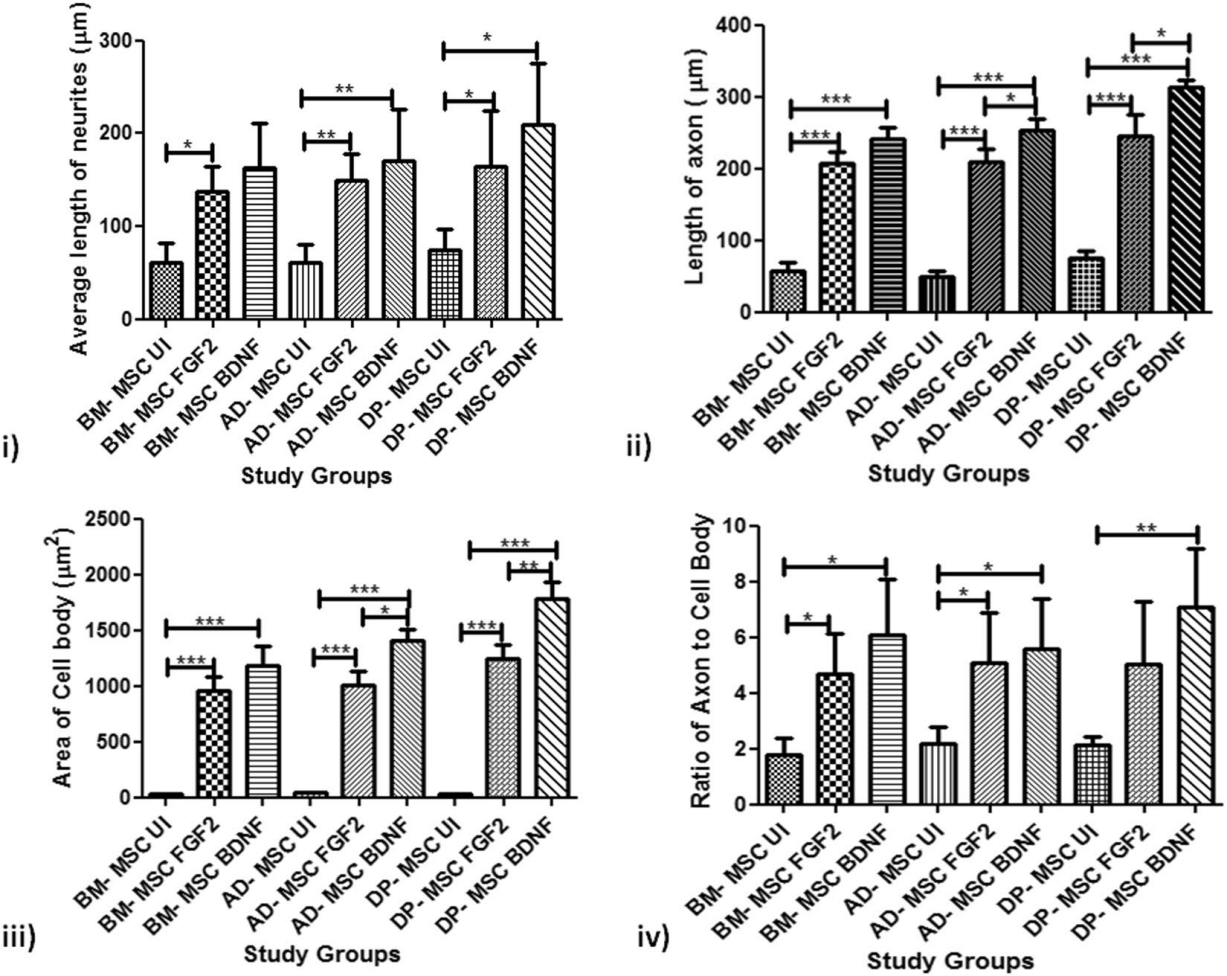
Morphometric Analysis of the neuronal cells generated by different induction protocols from various hMSC types. (i) Average length of neurites of cells under various study groups. The graph shows that the difference in the length of neurites is significant between both the induction protocols with all hMSC types, except in BM-MSC and AD-MSC induced with both induction protocols; (ii) Length of axons of cells under various study groups. The graph shows that the difference in the axonal length is non- significant between both the induction protocols with all hMSC types; (iii) Area of the cell body under various study groups. The graph depicts significant difference in this parameter between AD-MSC differentiated by two induction protocols (p*) and BM-MSC and DP-MSC induced with FGF2 and BDNF both (p**); (iv) Ratio of axon to cell body of cells under various study groups. The graph depicts that this morphological parameter of neironal cells shows significant difference in the two differentiation protocols followed on each hMSC type (p*). However, no significant difference was observed between various hMSC types. For all the parameters under the study, five different samples of each type of hMSC were taken (n = 40 for each study group). Data was analysed by three independent observers.

Morphological features of a neuronal cell are stated by the presence of distinct cell body, neuritic outgrowths and a long axon. Axon hillock-like structures were also observed on the cell bodies at the points from where neurites were found emerging. Upon morphometric analysis of the neurons generated under culture conditions using FGF2 and FGF2+ BDNF, it was observed that the diameter of cell body of induced cells was higher that that of respective uninduced hMSC. The area of CB increased from an average of 39.79 µm^2^ in uninduced (UI) hMSCs to 1072.73 µm^2^ in FGF2 induced and 1456.67 µm^2^ in hMSCs induced with FGF2 and BDNF both. The average neurites in hMSCs when induced with both the induction cocktails were significantly longer than those observed in respective uninduced hMSCs. Uninduced hMSCs didn’t develop neurite outgrowth. Hence, the distance from the cell body to the cell terminal was compared between the various study groups. The average neurites’ length increased from 65.38 µm in UI-hMSCs to 150.9 µm in FGF2 induced and to 180.9 µm in hMSCs induced with both FGF2 and BDNF. Similar trend was observed when the average length of axon was considered. The axonal length increased from an average of 60.76 µm in UI-hMSCs to 220.7 µm in FGF2 induced and to 268.3 µm in hMSCs induced with both FGF2 and BDNF. The ratio of axonal length and cell body diameter was also more than 3 in cells of all the induction groups. The ratio of axon and the diameter of the cell body in uninduced hMSCs was less than 2 while that in induced hMSCs ranged from 3–11. Detailed morphometric analysis is given in Fig. 2.

#### DP-MSC show higher upregulation of neuronal genes as compared to those in BM-MSC and AD-MSC

The expression of nestin, NF, MAP2, and TH genes was evaluated by RT-PCR in tissue specific hMSCs derived DA neuronal cells and respective uninduced hMSCs as experimental control (data not shown). Cells from both the induction groups showed upregulation of these genes. However, the expression of genes associated with mature DA neurons was higher in DP-MSC derived neuronal cells as compared to those derived from BM-MSC and AD-MSC.

**Figure 3:**
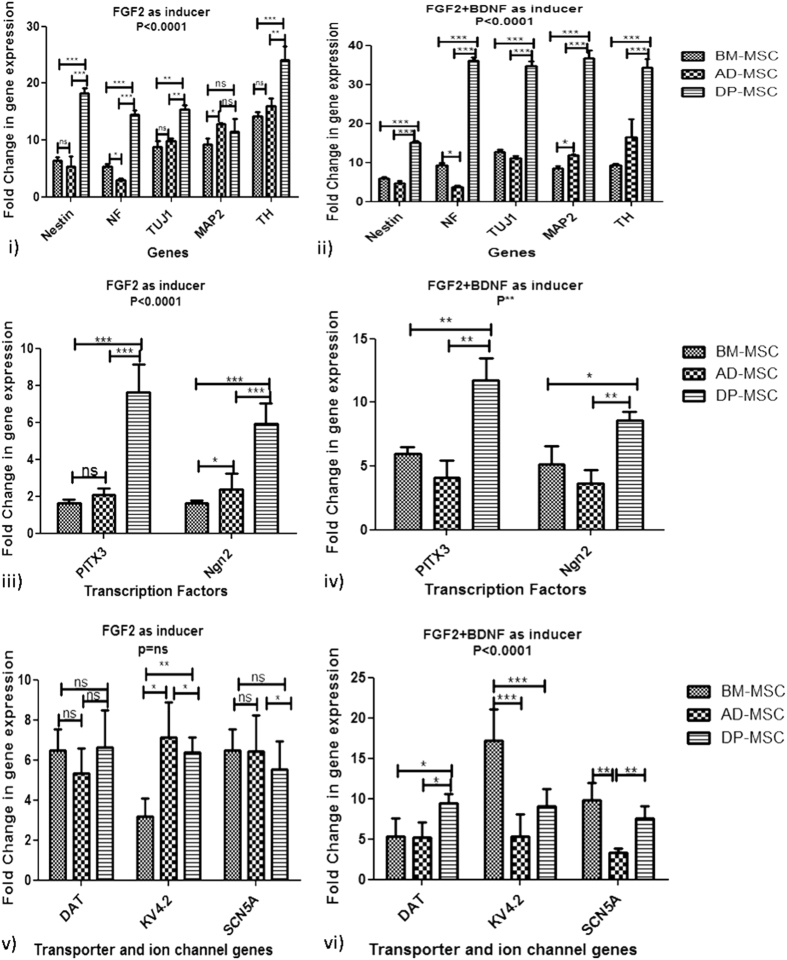
qRT- PCR gene analysis of differentiated hMSCs. (i) Relative fold change in neuronal gene expression in hMSCs induced with FGF2 alone; (ii) Relative fold change in neuronal gene expression in hMSCs induced with FGF2 and BDNF; (iii) Relative fold change in gene expression of transcription factors associatedwith DA neuron survival and proliferation in hMSCs induced with FGF2 alone; (iv) Relative fold change in gene expression of transcription factors associated with DA neuron survival and proliferation in hMSCs induced with FGF2 and BDNF; (v) Relative fold change in gene expression of ion channels and transporter protein in hMSCs induced with FGF2 alone; (vi) Relative fold change in gene expression of ion channels and transporter protein in hMSCs induced with FGF2 and BDNF.

The upregulation of neuron lineage-specific genes was further quantified as compared with their basic level expression in respective UI-hMSCs. On quantitative real time PCR analysis for Nestin, NF, TUJ1 and MAP2, it was found that fold change in the expression of these genes was much higher in induced DP-MSC (18.2 ± 0.434, 14.45 ± 0.328, 11.53 ± 0.96 and 15.38 ± 0.35 change folds with FGF2 and 15.2 ± 0.1906, 36.04 ± 0.4385, 36.71 ± 0.9258 and 34.82 ± 0.495 change folds with both FGF2 and BDNF, respectively) than that observed in case of induced BM-MSC or AD-MSC (Fig. 3i and ii). Similar trend was observed when the induced cells from all the hMSC types were analyzed for DA neuron specific genes, TH (14.22 ± 0.329 and 9.34 ± 0.152 change folds in BM-MSC, 15.99 ± 0.5805 and 16.47 ± 2.116 folds in AD-MSC and 24.17 ± 1.07 and 34.24 ± 0.985 change folds in DP-MSC induced with FGF2 and FGF2 + BDNF, respectively) and transcription factors Ngn2 (1.624 ± 0.06 and 5.16 ± 0.64 change folds in BM-MSC, 4.04 ± 0.689 and 3.656 ± 0.465 change folds in AD-MSC and 1.528 ± 0.1604 and 8.604 ± 0.299 change folds in DP-MSC induced with FGF2 and FGF2 + BDNF, respectively) and PITX3 (1.61 ± 009 and 5.97 ± 0.225 change folds in BM-MSC, 2.07 ± 1.692 and 4.105 ± 0.589 change folds in AD-MSC and 7.616 ± 0.6860 and 11.73 ± 0.78 change folds in DP-MSC induced with FGF2 and FGF2 + BDNF, respectively) (Fig. 3i–iv). However, fold change in the expression of functionality related genes, DAT, Kv4.2 and SCN5A did not follow the same trend as other genes under study (Fig. 3v and vi). There was no significant difference observed between the two induction protocols with BM-MSC and AD-MSC in the expression of DAT. Upregulation of Kv4.2 was maximum in BM-MSCs (17.17 ± 1.76 change folds), followed by DP-MSCs induced wth FGF2 + BDNF (9.058 ± 0.9528 change folds). Expression of SCN5A gene followed similar trend as Kv4.2 with maximum gene upregulation in BM-MSC (9.784 ± 0.9708 change folds), followed by DP-MSC induced wth FGF2 + BDNF (7.576 ± 0.6663 change folds).

#### Flow cytometric Analysis: (i) DP-MSC yield higher number of neuronal cells as compared to BM-MSC and AD-MSC

**Figure 4:**
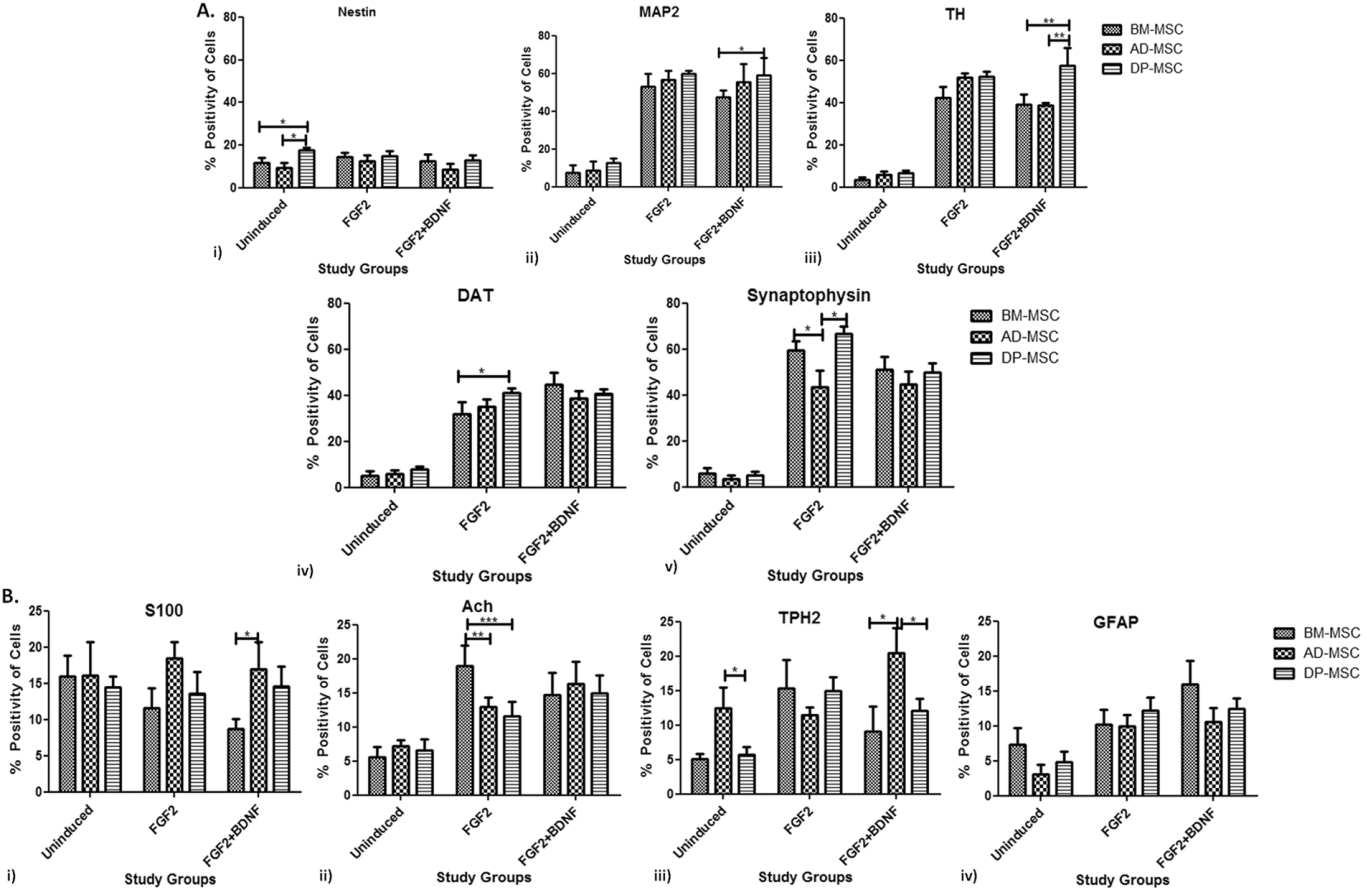
Flow cytometric analysis for cell enumeration: (**A)** (i) Graph depicting the number of nestin positive cells pre- and post- induction. DP-MSC have maximum number of nestin positive (p*), cells at the beginning of experiments, followed by those in BM-MSC and AD-MSC; (ii) Graph depicting the number of MAP2 positive cells pre- and post- induction. No significant difference was observed in various induction groups, except in DP-MSC induced with FGF2 and BDNF both (p*); (iii) Graph depicting the number of TH positive cells pre- and post- induction. No significant difference was observed in various induction groups, except in the two protocols with DP-MSC (p*); (iv) Graph depicting the number of DAT positive cells pre- and post- induction. No significant difference was observed in various induction groups, except in BM-MSC induced with FGF2 and BDNF both (p*); (v) Graph depicting the number of synaptophysin positive cells pre- and post- induction. No significant difference was observed in various induction groups, except between hMSCs induced with FGF2 alone (p*). Synaptophysin positive cells were least in induced AD-MSC with both the induction protocols. (**B**) (i) Graph depicting the number of S100 positive cells pre- and post- induction. AD-MSC induced with FGF2 alone have maximum number of S100 positive (p*); (ii) Graph depicting the number of Ach positive cells pre- and post- induction. No significant difference was observed under various study groups; (iii) Graph depicting the number of TPH2 positive cells pre- and post- induction. No significant difference was observed under various study groups, except in AD-MSC induced with FGF2 and BDNF both (p**); (iv) Graph depicting the number of GFAP positive cells pre- and post- induction. No significant difference was observed under various study groups, except in BM-MSC induced with FGF2 both (p*). Efficiency of both the protocols was also determined by this experimental analysis.

Flow cytometric analysis indicated that while the expression of Nestin in all the three hMSC types did not change significantly pre and post induction with any of the induction protocols (ranged from 6.92 ± 2.11% to 14.97 ± 1.01%); the expressions of MAP2 (ranged from 7.48 ± 1.78% in UI-hMSCs to 63.08% in differentiated hMSCs) and TH (ranged from 3.3 ± 0.54% in UI-hMSCs to 59.28 ± 3.07% in differentiated hMSCs) changed significantly in induced cells, proving the successful differentiation of hMSC into neuronal cells, with the highest percentage positivity of cells in DP-MSC induced neuronal cells. When both the induction protocols were compared, no significant difference in the expression of MAP2 and TH was observed, except in the study group where DP-MSC were induced with FGF2+ BDNF showed significantly higher percentage of TH positive cells. Detailed data is shown in the Fig. 4Ai–iii.

It was also observed that the percentage of cells expressing DAT and synaptophysin proteins increased significantly higher in differentiated hMSCs, as compared to the respective undifferentiated hMSCs. Percentage of DAT positive cells increased from 5.4 ± 0.9% in UI-hMSCs to as high as 49.6 ± 2.5% post-differentiation. Cells positive for synaptophysin were observed to have increased from 3.4 ± 0.6% to 61.40 ± 2.38%. While there was no significant difference observed in the percentage of cells positive for DAT (except between BM-MSC and AD-MSC induced with FGF2 alone), there was a decrease in the cells positive for synaptophysin in the induction protocol where BDNF was also used. This was the common observation with all the three hMSC types, used in this study (Fig. 4Aiv and v).

### After induction period, a mixed cell population was observed in the culture, comprising of glial cells, schwann cells, serotonergic and cholinergic neuronal cells apart from DAergic neurons

To investigate the different types of cells present in the cell culture after induction, flow cytometric enumeration was done, using cell type specific markers. Following data analysis, it was observed that after induction, cells positive for GFAP, ACH, TPH2 and S100 were present. However, the percentage of these cells was too low (Fig. 4Bi–iv) as compared to DAergic neuronal cells. While no significant difference was observed in the cells positive for S100 after induction in AD-MSC and DP-MSC, BM-MSC showed decrease in S100 positive cells when induced with FGF2 + BDNF. Percentage of ACH positive cells were observed to have increased in all the hMSC types with both the induction protocols (maximum positivity in BM-MSC induced with FGF2 alone). The percentage of serotonergic neuronal cells was found to be significantly higher when AD-MSC were induced with FGF2 alone (p*), as compared to the rest of the study groups. The difference between the percentage of the non-DAergic neuronal cell types was non- significant among the various study groups.

#### Differentiated DP-MSC had higher expression of neuronal cell specific proteins than BM-MSC and AD-MSC

**Figure 5:**
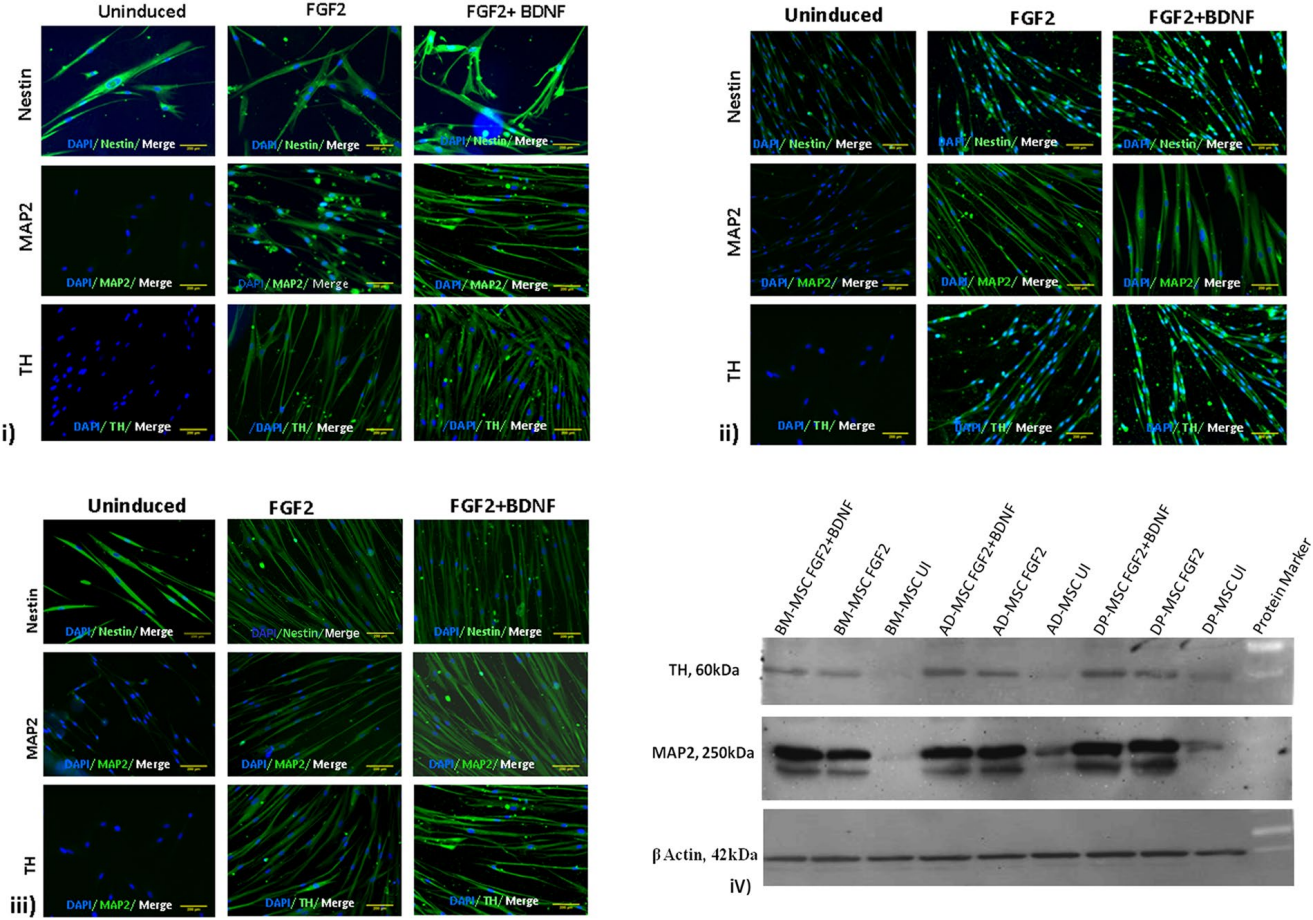
Immunoflorescence assay showing the expression of neuronal cell associated proteins in differentiated hMSCs. (i) BM-MSC; (ii) AD-MSC; (iii) DP-MSC. Expression of neuronal cells and DAergic neuronal cells specific protein has increased post- induction in all hMSC types. Also, DP-MSC are showing maximum expression of both MAP2 and TH proteins; (iv) Immunoblotting Assay for expression of neuronal and DA neuronal cells associated proteins (MAP2 and TH) in uninduced and differentiated hMSCs by both induction protocols.

After termination of neuronal differentiation period, hMSCs derived DA neuronal cells were labeled for neuronal specific proteins like nestin, MAP-2, TH, DAT and synaptophysin. Cells from all the study groups were found to be positive for these markers as compared to uninduced groups. There was minimal expression of MAP2 & TH in UI-hMSCs, while an upregulated expression was observed post-induction in all the hMSC types (Fig. 5i–iii). Increased fluorescence intensity in the images support higher expression of MAP2 and TH in differentiated cells. However, among the various hMSCs types, highest fluorescence intensity of these protein markers was observed in case of DP-MSC.

### Expression of neuronal and dopaminergic neuronal proteins in induced DP-MSC is higher than that expressed in induced BM-MSC

Immunocytochemistry examination showed cells positive for the neuronal markers, Nestin, MAP2 and TH in FGF-2 induced cells, as compared to uninduced hMSCs. Expression of MAP2 was observed at basal level in both BM-MSC, AD-MSC and DP-MSC while TH was not expressed at all in any of the three MSC types.

Translation of MAP2 and TH genes to form protein was more evident in case of DP-MSC as compared to that in BM-MSC and AD-MSC, post induction with both the induction protocols. This data is in accordance with that received by flow cytometric analysis of MAP2 and TH positive cells in induced and uninduced hMSCs (Fig. 5iv). The immunoblotting assay data when combined with that of flow cytometry for expression of MAP2 and TH, reveals that number of cells expressing MAP2 and TH is higher with DP-MSC as starting cell model, ensuring it as a better candidate to study neurogenesis under *in vitro* conditions.

### Functional Characterization of neuronal cells generated from BM-MSC, AD-MSC and DP-MSC

#### Synaptic plasticity of neuronal cells obatined from DP-MSC is higher as compared to that of BM-MSC

**Figure 6:**
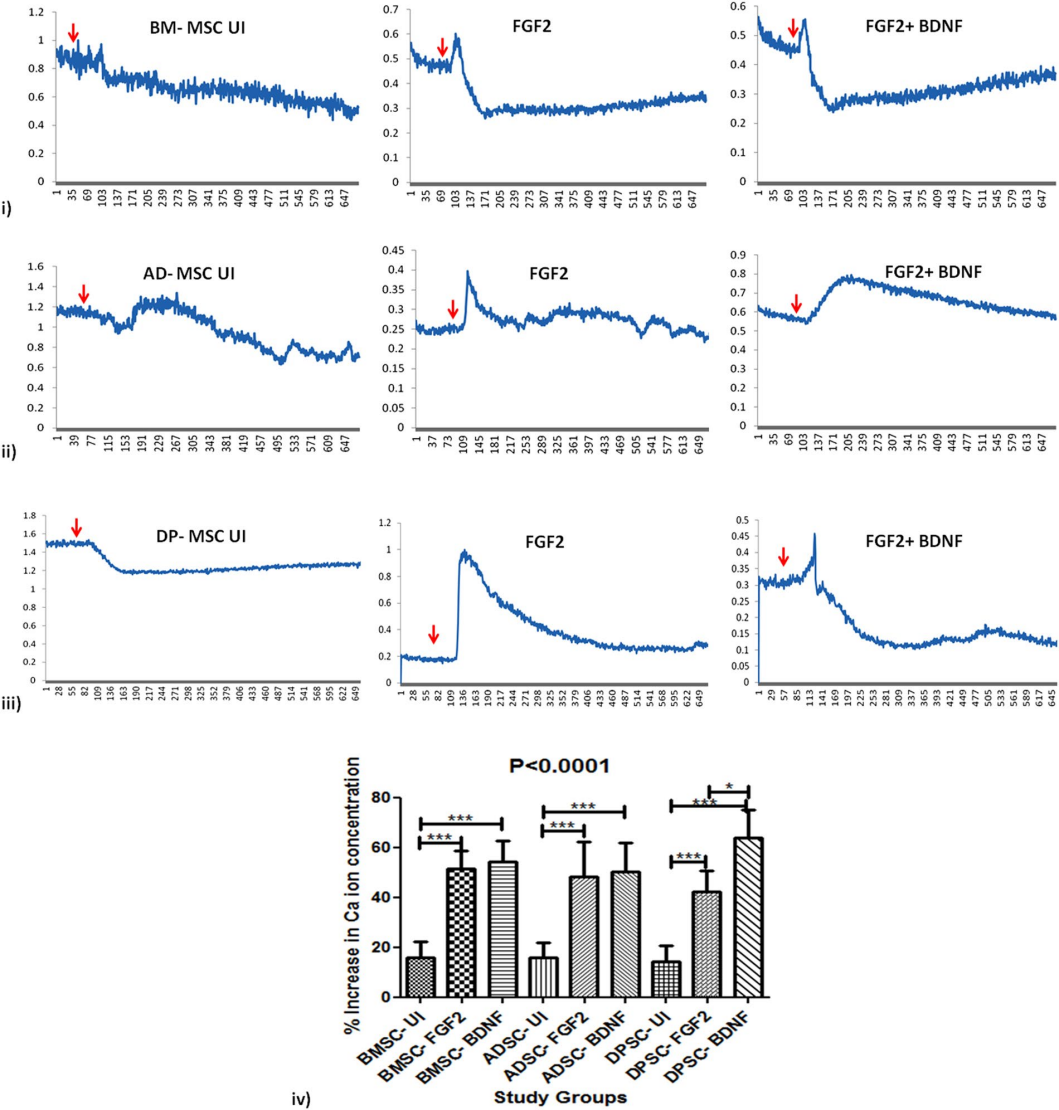
Calcium ion imaging analysis by Fura red- AM ratiometric dye. (i–iii) Line graphs showing the changes in the Ca^2+^ transients upon depolarization with KCl in cells of all study groups. The uninduced hMSC did not show any change in the intracellular Ca^2+^ concentration after depolarization. The red arrows indicate the time point of addition of KCl for depolarization in the cell culture; (iv) Graph showing the change in the percentage increase in the calcium ion concentration in hMSCs obtained from various tissue sources after neuronal induction by different protocols.

Results obtained suggest that induced hMSCs, upon treatment with 50 mM of KCl, show change in the calcium ion concentration in the cytosol of the neurons generated *in vitro*. While there was significant difference in the intracellular calcium ion transients in neuronal cells generated by both the induction protocols and all the three hMSC types, as compared to that in respective uninduced MSCs there was no significant difference observed between various hMSC types in both the induction protocols. The basal level of calcium ion efflux was 14.41 ± 1.399% in UI-hMSCs. this percentage change in calcium ion concentration reached as high as 63.83 ± 2.46% in DP-MSC induced with FGF2 + BDNF. The change in the calcium ion concentration in various study groups have been detailed in the Fig. 6i–iv (also see supplementary materials for respective videos).

## Discussion

Dopaminergic neurons are the sub- set of the neuronal cells which reside in the substantia nigra par compacta region of the mid brain and are capable of secreting dopamine as neurotransmitter. These neurons are important for neuro- muscular coordination. Degeneration of dopaminergic neurons is the main cause of Parkinson’s disease (PD). Several studies have been carried out towards cell based treatment for PD at both basic and translational levels^18, 22, 23^. To substantiate cell based treatment, it is crucial to understand the process of neurogenesis and dopaminergic neurogenesis. Several neurotrophic factors like BDNF, GDNF, neurotrophin-3 and nerve growth factor have been reported to generate mature DAergic neurons from human BM-MSC *in vitro* ^18^. According to this study, BDNF was most effective in generating mature functional DAergic cells, as evidenced by calcium ion imaging and electrophysiological properties of neuronal cells generated *in vitro*. According to our recently published study^18^, we have reported the generation of DAergic neurons by using a minimal concentration of only FGF2 (10 ng/ml). However, functionality of the neuronal cells was sub-optimal as compared to the already reported studies. The genes and proteins contributing towards the functionality of the neuronal cells were also not investigated. Therefore, the current study was designed to investigate the effect of BDNF in generating mature functional DAergic neurons with our previously reported protocol in terms of expression of genes, proteins, transcription factors and functionality of neuronal cells by calcium ion imaging. Through this study, we also aim to investigate the differential neuronal differentiation potential of tissue specific hMSCs and find the most optimal hMSC type for various kinds of basic and translational studies.

hMSCs can be differentiated into cells of neuronal lineage using FGF-2 and BDNF as the inducers^18, 20, 24, 25^. On differentiating tissue specific hMSCs by both the induction protocols, morphological changes were observed during the course of differentiation. Nucleus of the cells shifted towards periphery, showing the perikaryl feature of the neuronal cells. Multiple neurites (2–6) were observed emerging from the nucleus. Distinct and long axon was also found emerging from the axon hillock. The typical structure of neurons was observed with the ratio of axon length and diameter of cell body being more than three^26^. The cytoskeletal condensation or rearrangements occuring during the course of neurogenesis, leads to neuritogenesis i.e., neurite branching and outgrowth. The subsequent maturation of one neurite to form the longest neurite or axon is termed as axonogenesis. In the processes of neuritogenesis and axonogenesis, multiple cytoskeletal proteins are involved, which include intermediate filaments like neurofilament, microtubules like β- tubulin and actin filaments^27^. When both the induction protocols were compared in hMSCs, it was observed that average neurites were longer when BDNF was added in the induction cocktail. However, when the tissue specific hMSCs were compared, it was observed that DP-MSC had longest neurites with both the induction protocols.

The average length of axons in various hMSCs types and area of the cell body, were also following the same pattern as that of the neurites’ length. According to the previously published studies^26, 28^, the ratio of axon and cell body in neurons should be equal to or more than three. All the induction groups fulfilled this criterion of neuronal morphology. When both the protocols were compared, a higher ratio of axon and cell body was observed when BDNF was added in induction cocktail. This observation was consistent in all the hMSC types. These results clearly state that BDNF leads to morphological maturity of the DAergic neurons. The difference in neurites’ length between all the hMSC types may be attributed to the difference in their origin, as DP-MSC^29^ are neuro-ectodermal in origin, while both BM-MSC and AD-MSC^30^ are mesodermal in origin.

Differentiated hMSCs were characterized at transciptional and translational levels. qRT- PCR results show remarkable fold change in the gene expression of various genes related to neurons and dopaminergic neurons like nestin, neurofilament, MAP2, TUJ1 and TH. Apart from these genes, the differentiated cells were studied for the expression of genes representing transcription factors like Pitx3, responsible for specification and terminal differentiation of mesencephalic DAergic neurons and NGN2 which is responsible for maturation of DAergic neurons. Genes responsible for expression of channel ion proteins like Kv4.2 and SCN5A were also studied, along with DAT, a protein involved in packaging and transport of dopamine and synaptophysin, which is required for synapse formation between two communicating neuronal cells. Among all the tissue specific hMSCs taken under this study, DP-MSC faired better than other hMSC types in the upregulation of these genes with both the induction protocols (significant p value). However, when both the induction protocols were compared, it was observed that adding BDNF led to higher upregulation of DA neuronal associated genes except that in AD-MSC, where there was no significant difference in gene expression between both the induction protocols. These results were supporting those of the morphological analyses.

Differentiated neuronal cells, alongwith their respective control groups were characterized by immunoflorescence for the expression of nestin, MAP2 and TH proteins. It was observed that nestin was being expressed at baseline level in all the uninduced hMSCs and its expression did not change significantly post induction. Also, MAP2 was observed to be minimally expressing in uninduced hMSCs and its expression has increased remarkably post-induction. However, no expression of TH was observed in uninduced hMSCs. After induction, the expression of TH was high in all the hMSC types. While the differential expression of nestin, MAP2 and TH proteins in all hMSCs types induced with both the differentiation protocols was not significant it was remarkably higher in case of DP-MSC induced with FGF2 and BDNF as compared to that in BM-MSC and AD-MSC. These results were also confirming our previous experiments of morphological and transcriptional characterization of neuronal cells. One of the plausible reasons behind this could be the predisposition of DP-MSC towards cells of neuronal lineage attributed by their neuro-ectodermal origin^31^, as the baseline gene expression of nestin in DP-MSC was higher than that in the remaining hMSCs.

It was observed from the flow cytometric characterization of hMSCs that the percentage of MAP2 positive cells was highest in DP-MSC induced with FGF2 alone followed by that in BM-MSC and AD-MSC. In the induction set up,where BDNF was also added, no significant difference in the efficiency of generation of mature MAP2 positive neuronal cells with BM-MSC was observed, while the difference was significant with rest two hMSC types. However, DP-MSC showed higher number of TH positive cells as compared to those in BM-MSC and AD-MSC. Likewise, in terms of efficiency of generation of dopaminergic neurons (calculated by counting TH positive cells by flow cytometry), it was observed that the generation of TH+ cells was highest in DP-MSC (followed by that in AD-MSC and BM-MSC, with no significant difference between BM-MSC and AD-MSC. Similar results were observed when BDNF was added in the induction cocktail, i.e., DP-MSC yielded higher number of TH+ neurons as compared to that in AD-MSC and BM-MSC with no significant difference between BM-MSC and AD-MSC. These results shows that BDNF has significant effect on the generation of neurons or DAergic neurons, specifically in case of DP-MSC. The percentage of TH positive cells reported by our group is much higher than in the recently reported study with 14.49% TH positive cells only in human DP-MSC upon induction^32^.

Upon enumeration of presence of non- DAergic cells, i.e., cells which do not express DAergic neuronal marker, TH, it was observed that after induction the culture contained glial cells, schwann cells, serotonergic and cholinergic neuronal cells. However, the percentage of these cell types was minimal, and significantly lower than TH positive DAergic neuronal cells, in each study group. The percentage of GFAP positive cells was significantly higher in BM-MSC induced with FGF2 and BDNF (p*), as compared to all other study groups. This observation is crucial as GFAP positive cells help in survival and maintenance of DAergic neuronal cells. This fact can be harnessed for cell based treatment, as long-time survival and in turn, engraftment of the transplanted neuronal cells is expected. To the best of our knowledge, none of the *in vitro* hMSC differentiation studies have been reported targetting the presence of other cell types in the culture. After studying the functionality of the *in vitro* generated neurons by calcium ion imaging, it was observed that the calcium ion efflux was highest in DP-MSC followed by that in BM-MSC and AD-MSC when induced with FGF2 alone. However, the percentage change in calcium ion concentration upon KCl treatement was highest in DP-MSC followed by that in BM-MSC and AD-MSC with BDNF in induction cocktail. There was significant increase in the change in Ca^++^ concentration when hMSCs were induced with BDNF also in the induction media, with all the hMSCs. These studies also reveal that BDNF has additional effect on the generation of functional dopaminergic neurons from hMSCs, with maximal effect on DP-MSC. However, there is no differential effect on the calcium ion efflux of BDNF on BM-MSC and AD-MSC. Our results are in concordance with other reported studies^18, 22, 33^.

This study is in accordance with the other studies in terms of characterization of neuronal cells derived from the three cell sources being used in the current study^18, 20–22, 34^. This study is the first of its kind, where we have not only compared the differentiation potential of various tissue specific hMSCs, but also the effect of different induction protocols on the same platform. As per our knowledge, no study has reported the comparative analysis of potential of hMSCs obtained from various human tissue sources, towards generation of functional dopaminergic neurons *in vitro*. Not only do we propose the cost-effective induction protocol with minimum adjuvents or inducers, we also report the most optimum hMSC tissue source for generating functional DA neurons *in vitro*. This study gives an insight into the response of various hMSCs towards various neuronal induction protocols. Unlike several other studies, this study substantiates the results and outcomes on primary cells obtained from five different donors for each tissue type.

To summarize, DP-MSC are equivalent to BM-MSC and AD-MSC in terms of expansion and expression of MSC surface markers. However, DP-MSC yield better cells in terms of expression of genes and proteins specific to neurons and DAergic neurons. They could be a better candidate for transplantation in regenerative medicine and study of neurogenesis due to their easy accessibility, proliferation rate, differentiation potential and origin (as DP-MSC are neuro-ectodermal in origin and initial concentration of nestin positive cells was significantly higher as compared to other hMSCs under study). Considering results of the two induction media cocktails used in the study, it may be also be concluded that BDNF has significant effect on the generation of functional dopaminergic neurons as compared to only FGF2. The results were consistent with all the tissue specific hMSCs. Our study also provides evidences that DP-MSC may be successfully used in transplantation studies and prove to be a better cell model to study neurogenesis, neuritogenesis, axonogenesis and functionality of the DAergic neurons or in pharmacological testing of various drugs.

These days the regenerative capacity of AD-MSC is being explored extensively in clinical trials for varied diseases and their regenerative potential is considered as equivalent to that of BM-MSC^6, 35^. Our study is in accordance with this concept, as throughout the study, BM-MSC and AD-MSC were almost equivalent to all the analytical parameters. However, DP-MSC showed maximum efficiency of generation of functional DA neurons at transcriptional, translational and functional levels. They may be taken as better cell model to study neurogenesis in future or for drug testing studies. This behaviour of DP-MSC may be attribiuted to their origin from neural crest, having better propensity of differentiation towards cells of neuronal lineage^36^. Our present study needs further validation by pre-clinical transplantation studies to comment on the safety and efficacy of transplantation of hMSC in Parkinson’s disease animal models.

## Materials and Methods

The study was commensed after getting ethical clearance from Institute Review Board (IRB) and Institutional Committee for Stem Cell Research (IC-SCR), AIIMS, New Delhi. All the methods described in this study were performed in accordance with the relevant guidelines and regulations of the Institution.

### Cell Culture: Revival and expansion of Bone Marrow- Mesenchymal Stem Cells (BM-MSC), Adipose tissue derived Mesenchymal Stem Cells (AD-MSC) and Dental pulp derived Mesenchymal Stem Cells (DP-MSC)

Cryopreserved BM-MSC, AD-MSC and DP-MSC were used for the study. Informed consent was obtained from the patients or legal representatives at the time of bone marrow, adipose tissue or extracted tooth/teeth collection for previous research projects. Age of patients ranged from 24 to 40 years for both BM-MSC and AD-MSC and 20–28 years for DP-MSC. Cells of 5 healthy donors of each MSC type, which were cryopreserved at first passage, were revived in DMEM-LG medium (Gibco, USA) with 10% fetal bovine serum (FBS) (Hyclone, USA) and penicillin (100 U/ml) streptomycin (100 μg/ml) (Gibco, USA) (Expansion medium), expanded and characterized. Viability of the revived cells was measured by trypan blue staining (Invitrogen, Life Technologies, USA). Followed by characterization (by surface marker profiling using flow cytometry and trilineage differentiation potential), cells from 3^rd^ to 5^th^ passage were used for all the further experiments.

Growth and proliferation rate was assessed by MTT assay and population doubling time was also studied (Supplementary material).

#### Neuronal Differentiation

For neuronal differentiation, induction medium containing Neurobasal media (Gibco, USA), B27 supplement (Gibco, USA), EGF and FGF2 (10 ng/ml each) (PeproTech Asia), L-Glutamine (Gibco, USA) PenStrep (Gibco, USA) was used. The induction protocol was carried out for 12 days with media change on every 3^rd^ day. Another set of study group included induction of hMSCs using the above mentioned induction media cocktail for 8 days, followed by addition of BDNF (50 ng/ml) on 9^th^ day. After termination of induction period at 12 days in both the study groups, the cells were used for further experiments^18, 20^.

### Neurites’ length Analysis

Induced hMSCs were examined for the morphological changes under inverted microscope. Images were captured at 10X magnification and analysed using SI Viewer software (Tokyo, Japan) for the number and length of neurites, length of axon and area and diameter of the cell body. Uninduced respective hMSCs were used as experimental control.

### Transcriptional Characterization of MSC induced into neuronal cells: Quantitative reverse transcription- polymerase chain reaction (qRT-PCR)

After differentiation, total RNA from all the experimental groups was extracted by phenol-chloroform method as previously described^20^. Single strand cDNA synthesis was performed by using cDNA synthesis kit from Life Technologies (California, USA) according to the manufacturer’s protocol. Expression of Nestin, Neurofilament (NF), Microtubule associated protein (MAP2) and Tyrosine hydroxylase (TH) was studied in both induced and uninduced hMSCs. All these primers were obtained from Sigma (Missouri, USA) (data not shown).

qRT-PCR experiments were performed using Realplex real time PCR detection system (Eppendorf, Germany) as previously described^20^. qRT-PCR was done for Nestin, NF, MAP2, β III tubulin (Tuj1), TH and transcription factors, PitX3 and Ngn2. To study the genes related to dopamine transportation, expression of DAT (dopamine transporter) gene was studied. Apart from these, genes related to various ion channels like Kv4.2 (potassium channel) and SCN5A (sodium channel) were also studied. Primers of qRT- PCR grade were procured from Sigma (Missouri, USA).

The expression of the genes of interest was normalized to that of the housekeeping gene, glyceraldehyde-3-phosphate dehydrogenase (GAPDH). Melting curve was used to confirm the results and data were analyzed using the graph pad prism software. Details of the primers used is given in Table 1.

**Table 1:** List and details of primers used for relative gene analysis by qRT-PCR in pre- and post- induction human Mesenchymal Stem Cells.

S. No.	Gene	Primer sequence	Annealing Temperature	Amplicon size
1.	GAPDH	5′-GACAAGCTTCCCGTTCTCAG-3′	57.0 °C	198 bps
		5′-GAGTCAACGGATTTGGTCGTCGT-3′		
2.	Nestin	5′-GCCCTGACCACTCCAGTTTA-3′	57.5 °C	198 bps
		5′-GGAGTCCTGGATTTCCTTCC−3′		
3.	NF	5′-AGGGATCCAGGAAGGAAGA -3′	55.0 °C	117 bps
		5′-TGACAACGCCTTTCTCCTCT-3′		
4.	MAP2	5′-ACTCCCTCTACAGCTGAGCC-3′	55.2 °C	182 bps
		5′-TGCCCAAACAAAGCTAGGGG-3′		
5.	Tuj1	5′-GCAAGGTGCGTGAGGAGTAT-3′	60.9 °C	157 bps
		5′-TCGTTGTCGATGCAGTAGGT-3′		
6.	TH	5′-GGTCGCGCTGCCTGTACT-3′	53.9 °C	125 bps
		5′-TCATCACCTGGTCACCAAGTT-3′		
7.	Ngn2	5′-ACCACCACAACACACGAGAC-3′	62.0 °C	196 bps
		5′-TTGCTGTGACTTTGGCCTGT-3′		
8.	Pitx3	5′-GGAGTCTGCCTGTTGCAGGA-3′	59.9 °C	173 bps
		5′-CGAGGCCTTTTCTGAGTCGC-3′		
9.	DAT	5′-TCCAGTGGAGACAGCTCGG-3′	59.1 °C	174 bps
		5′-GCTGAAGTAGAGCAGCACGA-3′′		
10.	Kv4.2	5′-AGTATTTGCTTCGCCTGGCT-3′	60.4 °C	152 bps
		5′-TCGGAGTGTGACAAAGGCTC-3′		
11.	SCN5A	5′-CAGGCCTTTGACGTCACCAT-3′	56.7 °C	147 bps
		5′-GAGCACAGTGCCTGTGAAGAT-3′		

### Immunocytochemistry

The assay was performed as previously described^20^. Briefly, fixed cells were incubated overnight at 4 °C with primary monoclonal antibodies against Nestin (1:400), MAP2 (1:250) and TH (1:200) (Abcam, USA). After induction period, hMSCs were washed five times with PBS and incubated with fluoro isothiocyanate (FITC) conjugated secondary antibodies (1:500 dilution) for 1 hour at room temperature. Finally, after washing five tmes with PBS, cells were counter stained with 4′,6-diamidino-2-phenylindole (DAPI) to visualize the cell nuclei. Cells were washed thrice with PBS to remove excess DAPI stain. Stained cells were examined using a fluorescence microscope equipped with a digital camera (Nikon Eclipse 80i, Japan).

### Intracellular staining for Flow Cytometry

hMSCs after induction with both the induction protocols, were labeled for Nestin (1:100), MAP2 (1:200), TH (1:100), synaptophysin (1:100), DAT (1:100), GFAP (1:100), TPH2 (1:150), Ach (1:150) and S100 (1:100) as previously described^20^. Briefly, the cells were incubated with primary antibodies (dilution of 1:100) for 1 hour 20 mins at 4 °C, followed by washing and incubation with secondary antibody labeled with fluorochrome tagged secondary antibody (dilution of 1:400) for 30 mins at room temperature. The cells were then washed and suspended in PBS and acquired on BD LSR II flow cytometer (Becton Dickinson, USA) with minimum of 10,000 events for each sample and analyzed with FACs DIVA software (version 6.1.2). All the antibodies were procured from Abcam, USA.

### Immunoblotting

Immunoblotting for the expression of neuronal cell specific proteins was performed with both induced and uninduced control cells, as previously described^18^. Briefly, after preparing whole cell lysates using RIPA buffer (Sigma, USA), the protein quantification was done using Bicinchoninic Acid (BCA) Assay method. Protein extracts (30 μg) were subjected to SDS-PAGE using 12% Tris/HCl SDS (Sodium dodecyl sulphate) gels and transferred onto PVDF membranes (Membrane Technologies, India). After blocking the membranes with 3% BSA, they were incubated with primary antibody against β-actin (Abcam,UK, 1:2500), MAP-2 (Santa Cruz, USA, 1:1000) and tyrosine hydroxylase (Santa Cruz, USA, 1:1000) in 1% BSA- phosphate saline buffer (PBS) overnight at 4 °C. Post incubation, membranes were washed thrice in PBST and incubated with the appropriate horseradish peroxidase (HRP)- conjugated secondary antibody (1/4,000) (Dako, USA) for 2 hours at room temperature. Membranes were developed with chemiluminescence detection reagent (Pierce, USA) and acquired by using Gel Imager machine (Fluor Chem E, Cell Biosciences, Australia).

### Calcium ion Imaging

Change in the concentration of calcium ions was studied by calcium ion imaging in hMSCs induced for 12 days in all study groups, as previously described^18^. Briefly, hMSCs upon induction, were stained with 10 µM of Fura red AM dye. after washing thrice with HBSS, the cells were activated using 50 mM KCl solution. Time lapse recording was made at 488 nm and 457 nm for 3 minutes. Baseline readings were obtained before adding KCl soultion to the cells. The experiment was performed using Leica Confocal Microscope (Model TCS SP8). The ratio of florescence at both the wavelengths was obtained and respective graph was plotted. The experiment was performed on 3 samples each. The data was analysed using Leica LAS AF software.

### Data Interpretation and Statistical Analysis

Means ± SD of independent experiments were analyzed by student’s t-test, one way and two way ANOVA test (as per the requirement of data analysis). P < 0.05 was considered as statistically significant. Analysis of data was done by using GraphPad Prism 5.00 software (San Diego, California, USA).

## Electronic supplementary material

Calcium ion imaging: BM-MSC UI: Video showing the calcium ion transients after adding KCl in uninduced BM-MSC

Calcium ion imaging: BM-MSC FGF2: Video showing the calcium ion transients after adding KCl in BM-MSC induced with FGF2 alone

Calcium ion imaging: BM-MSC FGF2+BDNF: Video showing the calcium ion transients after adding KCl in BM-MSC induced with FGF2 and BDNF

Calcium ion imaging: AD-MSC UI: Video showing the calcium ion transients after adding KCl in uninduced AD-MSC

Calcium ion imaging: AD-MSC FGF2: Video showing the calcium ion transients after adding KCl in AD-MSC induced with FGF2 alone

Calcium ion imaging: AD-MSC FGF2+BDNF: Video showing the calcium ion transients after adding KCl in AD-MSC induced with FGF2 and BDNF

Calcium ion imaging: DP-MSC UI: Video showing the calcium ion transients after adding KCl in uninduced DP-MSC

Calcium ion imaging: DP-MSC FGF2: Video showing the calcium ion transients after adding KCl in DP-MSC induced with FGF2 alone

Calcium ion imaging: DP-MSC FGF2+BDNF: Video showing the calcium ion transients after adding KCl in DP-MSC induced with FGF2 and BDNF

Supplementary Material
