# Supplementary material for: Synergistic Effect of BDNF and FGF2 in Efficient Generation of Functional Dopaminergic Neurons from human Mesenchymal Stem Cells
Source: Sci Rep. 2017 Sep 4;7:10378. doi: 10.1038/s41598-017-11028-z (PMC5583182; doi:10.1038/s41598-017-11028-z)
Supplement: Supplementary file 10 — Supplementary Material [file 41598_2017_11028_MOESM10_ESM.pdf]

Synergistic Effect of BDNF and FGF2 in Efficient Generation of Functional Dopaminergic Neurons from human Mesenchymal Stem Cells

Authors:

Manisha Singh[1], Anupama Kakkar[1], Rinkey Sharma[1], O.P. Kharbanda[2], Nitika Monga[2], Manish Kumar[3], Shantanu Chowdhary[3], Balram Airan[4], Sujata Mohanty[1*]

Affiliations:

[1]Stem Cell Facility *(DBT- Centre of Excellence for Stem Cell Research)*, All India Institute of Medical Sciences, New Delhi, India

[2] Department of Orthodontics and Dentofacial Deformities, Centre for Dental Education and Research (CDER), All India Institute of Medical Sciences, New Delhi, India

[3]Institute of Genomics and Integrative Biology, New Delhi, India

[4]Department of Cardiothoracic and Vascular Surgery, All India Institute of Medical Sciences, New Delhi, India

Corresponding Author:

Prof. Sujata Mohanty
Stem Cell Facility *(DBT- Centre of Excellence for Stem Cell Research)*
All India Institute of Medical Sciences, New Delhi, India
Present Address: Stem Cell Facility
1st Floor, ORBO Complex
All India Institute of Medical Sciences,
Ansari Nagar, New Delhi, India-110029

Permanent Address: -do-

Tel: 91-9868398194/ 91-9810291336
Fax: 91-11-26588641/ 91-11-26588603
e-mail ID: drmohantysujata@gmail.com

Characterization and Proliferation Studies of human Mesencymal Stem Cells:

MSC Phenotyping:

Phenotyping of hMSCs at 3rd passage was performed as per the already standardized protocol of the lab (Nandy et al., 2014). Briefly, hMSCs were harvested using 1X TrypLE Express (Life Technologies, USA) and single cell suspension was prepared in staining buffer at a concentration of $1×10^5$ cells/ml. Cells were stained with anti- human CD73-PE, CD90-PECy5, HLA Class I-APC, HLA Class II-FITC (Becton Dickinson, USA), CD29- FITC and CD105- APC (eBioscience, USA). Unlabeled cells were taken as experimental control. The cells were acquired on BD LSR II flow cytometer (Becton Dickinson, USA) with a minimum of 5000 events for each sample and analyzed with FACs DIVA software (version 6.1.2)

Growth Kinetics Assay:

Cell Proliferation Assay (MTT Assay ((3-(4,5-Dimethylthiazol-2-yl)-2,5-diphenyltetrazolium bromide*))*

Cell proliferation rate was assessed on hMSCs at third passage as per the previously reported protocol of the lab (Nandy et al., 2014). Briefly, at 80-90% confluency, cells in T25 culture flasks (Becton Dickinson, USA) were trypsinized using TryplE (Gibco, USA) and harvested by centrifugation at 800 rcf for 5 minutes. Supernatant was aspirated and pellets were re-suspended in fresh expansion medium. After cell counting, hMSCs from each sample were plated in triplicates at a density of $5X10^6$ cells/well in a 96-well plate. Cell expansion media was changed every alternate day. Cell proliferation assay was done on day1, 3,6,9,12,15 and 22 of the culture, whereexpansion medium was replaced with 180µl fresh media and 20µl MTT ((a tetrazole), Sigma, USA) reagent (5mg/ml in PBS) and incubated for 4 hours at 37˚C in CO_2 incubator, allowing live cells to form formazan crystals. Post incubation period, media was aspirated and 150µl DMSO (Sigma, USA) was added

to each well in order to dissolve formazan crystals and incubated at 37˚C for 30 min in dark. Later, the supernatants were transferred to a separate flat- bottom 96- well plate and the optical densities were obtained at 570nm and 660nm using a spectrophotometer (BioTek, USA).

Population Doubling Time (PDT) Assay:

PDT for hMSCs were calculated by plating $1X10^4$ cells from 3rd passage per 35mm petri plate (Becton Dickinson, USA). The assay was performed in triplicates and incubated at 37˚C/5% CO_2. After obtaining 70-75% confluency, hMSCs were harvested, counted and assessed for viability (Trypan Blue dye exclusion). The PDT was obtained by the formula given below:

PDT= T−To Log2(LogN−LogNo)

Where, T: Time of harvesting

To: Time of seeding

N: Number of cells harvested

No: Number of cells seeded

Tri-lineage Differentiation:

hMSCs were characterized by differentiating them into tri- lineage, i.e., cells of osteogenic, chondrogenic and adipogenic lineages as per our previously published research article (Nandy et al., 2014).

Results:

Characterization and proliferation study of hMSCs:

Upon characterizing hMSCs obtained from the three tissue sources, i.e., bone marrow, adipose tissue and dental pulp by surface marker profiling using flow cytometry, it was observed that these hMSCs were positive for CD29, CD73, CD90,

CD105 and HLA class I, while they were found negative for hematological markers like CD34/45 and HLA class II, with no significant difference. Trilineage differentiation experiments also supported the results obtained from surface marker profiling, with exception of DP- MSCs showing low levels of differentiation into adipocytes (Supplementary Figure 1).

Proliferation rate (MTT assay) and population doubling time (PDT) of all the three hMSC types were also studied. It was observed that the PDT of DP- MSCs (36±3.5 hours) was significantly lower than that of BM-MSCs (40±2.8 hours) or AD- MSCs (40.90 ± 0.5686 hours). These results were further strengthened by proliferation assay of the three hMSC types under study (Supplementary Figure 2).

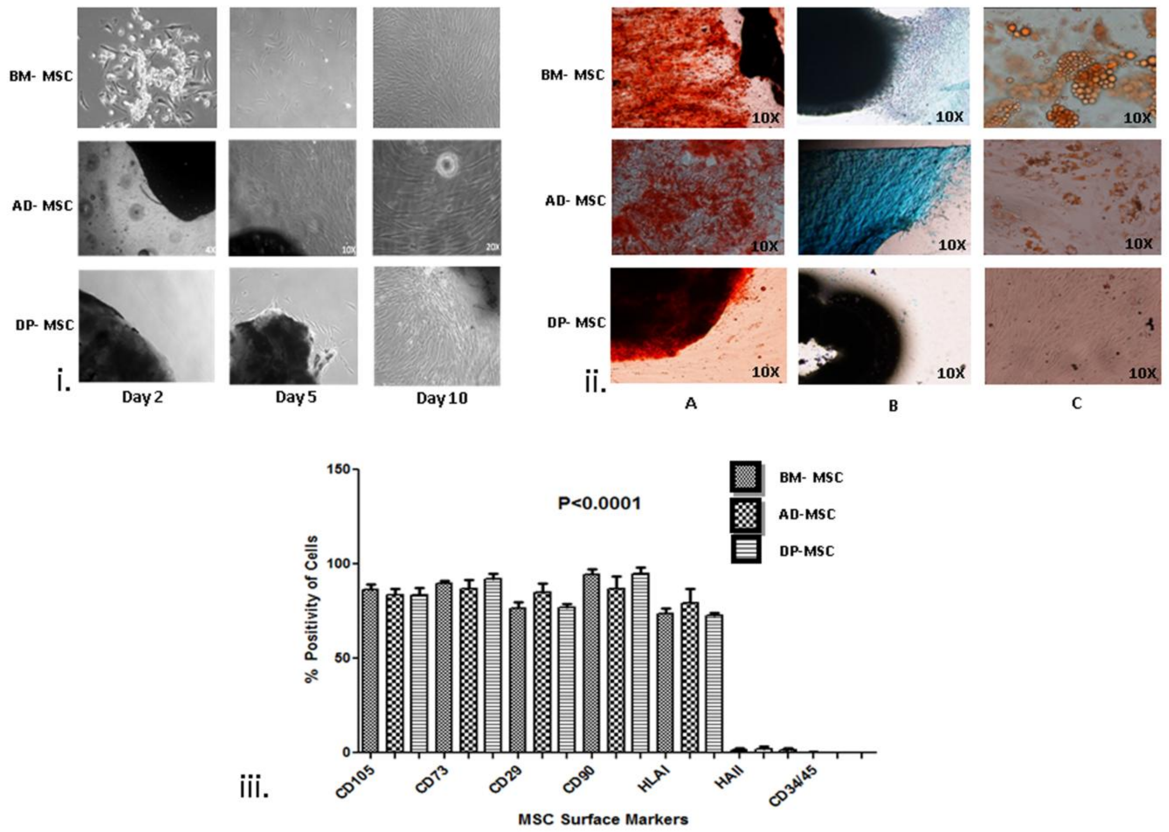

Supplementary Figure 1: Isolation, expansion, characterization of hMSCs. *i) Explant culture method to isolate MSCs from bone marrow, adipose tissue and dental pulp; ii) Tri-lineage differentiation of hMSCs into A. Osteocytes, B. Chondrocytes and C. Adipocytes; iii) Surface marker profiling of hMSCs by flow cytometry.*

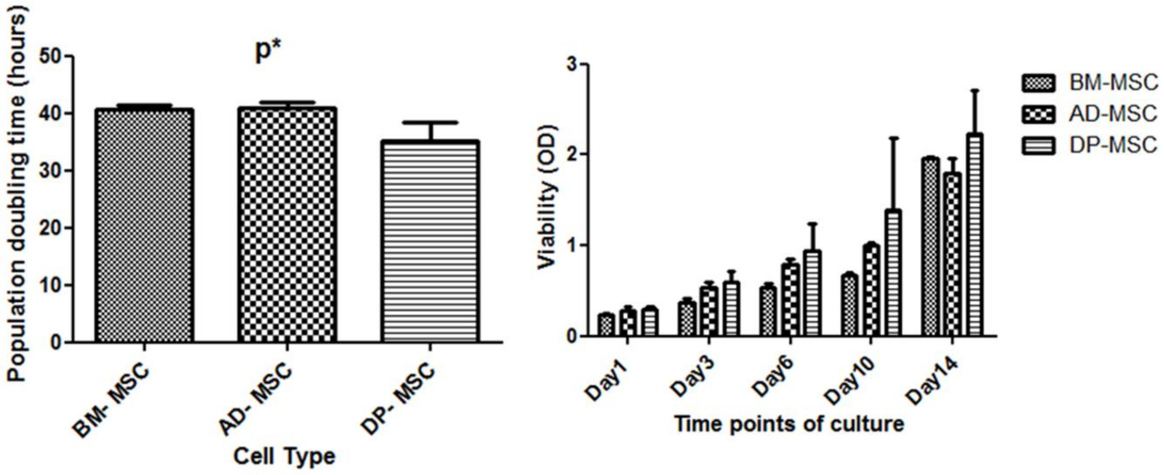

***Supplementary Figure 2: Proliferation assay of hMSCs** i) Population doubling time of hMSCs; ii) Proliferation assay of hMSCs by MTT assay*

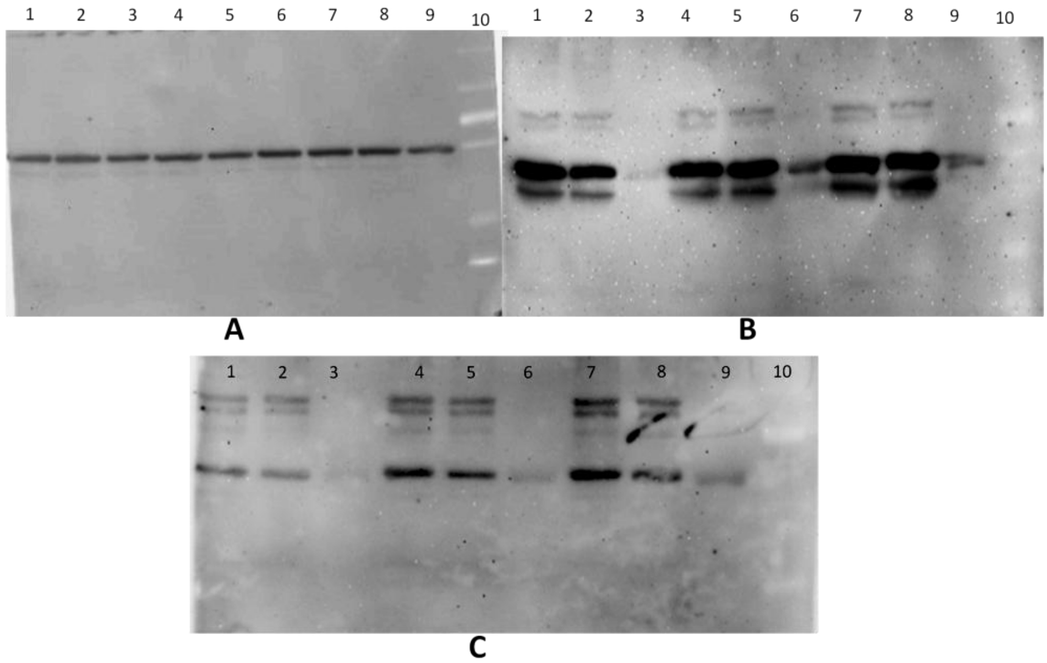

Supplementary Figure 3: Full length blot images of the immunoblotting assay: Pre- stained protein marker is shown in the starting of the blots. **A) β-actin, B) MAP2 and C) TH proteins on the blot**

Lane Number	Name of the protein sample
1.	BM-MSC FGF2+BDNF
2.	BM-MSC FGF2
3.	BM-MSC UI
4.	AD-MSC FGF2+BDNF
5.	AD-MSC FGF2
6.	AD-MSC UI
7.	DP-MSC FGF2+BDNF

8.	DP-MSC FGF2
9.	DP-MSC UI
10.	Protein marker

Video Legends:

Video 1: Calcium ion imaging: BM-MSC UI: Video showing the calcium ion transients after adding KCl in uninduced BM-MSC

Video 2: Calcium ion imaging: AD-MSC UI: Video showing the calcium ion transients after adding KCl in uninduced AD-MSC

Video 3: Calcium ion imaging: DP-MSC UI: Video showing the calcium ion transients after adding KCl in uninduced DP-MSC

Video 4: Calcium ion imaging: BM-MSC FGF2: Video showing the calcium ion transients after adding KCl in BM-MSC induced with FGF2 alone

Video 5: Calcium ion imaging: AD-MSC FGF2: Video showing the calcium ion transients after adding KCl in AD-MSC induced with FGF2 alone

Video 6: Calcium ion imaging: DP-MSC FGF2: Video showing the calcium ion transients after adding KCl in DP-MSC induced with FGF2 alone

Video 7: Calcium ion imaging: BM-MSC FGF2+BDNF: Video showing the calcium ion transients after adding KCl in BM-MSC induced with FGF2 and BDNF

Video 8: Calcium ion imaging: AD-MSC FGF2+BDNF: Video showing the calcium ion transients after adding KCl in AD-MSC induced with FGF2 and BDNF

Video 9: Calcium ion imaging: DP-MSC FGF2+BDNF: Video showing the calcium ion transients after adding KCl in DP-MSC induced with FGF2 and BDNF
